# Major Sensing Proteins in Pathogenic Fungi: The Hybrid Histidine Kinase Family

**DOI:** 10.1371/journal.ppat.1005683

**Published:** 2016-07-28

**Authors:** Anaïs Hérivaux, Yee-Seul So, Amandine Gastebois, Jean-Paul Latgé, Jean-Philippe Bouchara, Yong-Sun Bahn, Nicolas Papon

**Affiliations:** 1 Université d'Angers, Groupe d'Etude des Interactions Hôte-Pathogène, Angers, France; 2 Department of Biotechnology, College of Life Science and Biotechnology, Yonsei University, Seoul, Republic of Korea; 3 Unité des Aspergillus, Institut Pasteur, Paris, France; 4 Laboratoire de Parasitologie—Mycologie, Centre Hospitalier Universitaire d’Angers, Angers, France; Geisel School of Medicine at Dartmouth, UNITED STATES

## Milestones in the Discovery of Histidine Kinases

**Fig 1:**
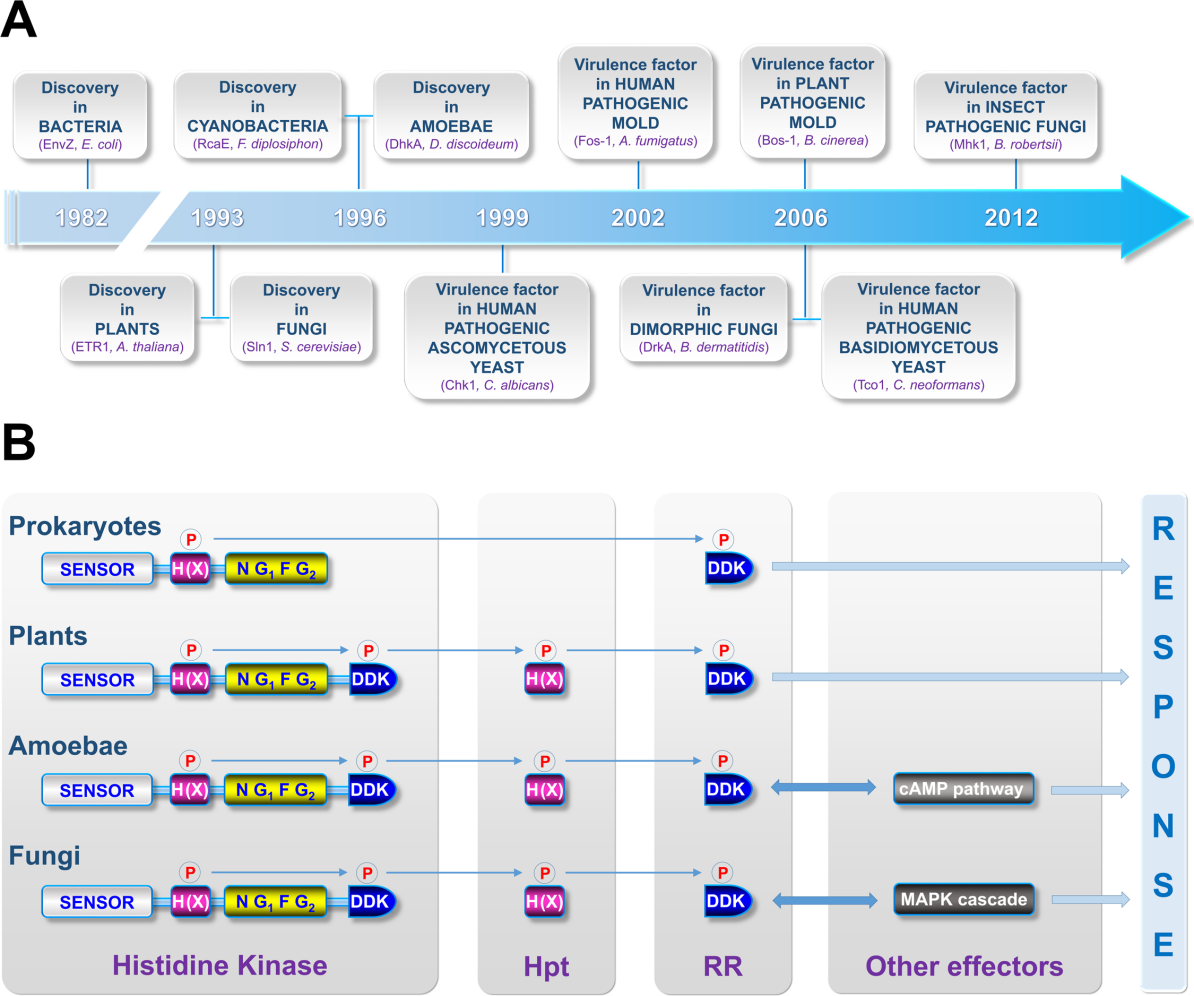
Milestones in the discovery of histidine kinases (HKs) and currently accepted canonical signaling pathways involving HKs in prokaryotes, plants, amoebae, and fungi. (A) Historical timeline depicting the evolution of knowledge concerning HKs. In the order of appearance from left to right: the EnvZ osmosensor in *Escherichia coli* [1], the phytohormone ethylene receptor ETR1 in *Arabidopsis thaliana* [2], the Sln1 osmosensor in *Saccharomyces cerevisiae* [3], the RcaE cyanobacteriochrome [5], the discadenine receptor DhkA in *Dictyostelium discoideum* [6], the quorum sensing-associated Chk1 in *Candida albicans* [26], the virulence factor Fos-1 in *Aspergillus fumigatus* [27], the dimorphism-related Drk1 in *Blastomyces dermatitidis* [28], the Bos-1 osmosensor in *Botrytis cinerea* [29], the *Cryptococcus neoformans* Tco1 and Tco2 (a first functionally characterized dual HK) [30], and the *Metarhizium robertsii* Mhk1 [31]. (B) Canonical schemes depicting signaling pathways involving HKs in prokaryotes, amoebae, plants, and fungi. In prokaryotes, most signaling pathways involving HKs simply consist of two components. The perception of a stimulus by the sensor domain (grey box) induces the autophosphorylation of a conserved histidine (H, pink box) by the catalytic domain (N G_1_ F G_2_, yellow box) in the HK. The phosphate is then transferred to a conserved aspartate residue (D) located on a cytosolic response regulator (RR) and the activated RR governs the expression of response genes. In plant cells, most (but not all) HKs constitute the initial sensing proteins of a four-step phosphorelay signaling pathway involving phosphorylation events of two downstream elements, i.e., histidine phosphotransfer shuttle proteins (Hpt) and RRs. Note that a first phosphorylatable receiver domain (DDK) is fused to the catalytic domain (N G_1_ F G_2_) in the HK. As observed for the archetypal two-component system in prokaryotes, the activated RR governs the expression of response genes. In amoebae, similarly to plants, a four-step phosphorelay signaling pathway is observed, but this latter controls a downstream cyclic AMP pathway. Finally, in fungi, knowledge is very fragmented, but initial studies in *Saccharomyces cerevisiae* have demonstrated that HKs also constitute the initial sensing proteins of a four-step phosphorelay signaling pathway that governs a cascade of mitogen-activated protein (MAP) kinases.

The pioneering discovery of histidine kinases (HKs) from *Escherichia coli* was made in the early 1980s with the identification of the *envZ* gene [1] (Fig 1A). Further biochemical characterization of the corresponding protein revealed a new type of protein kinase activity, namely HK, to add to the well-known serine/threonine and tyrosine kinases. For a decade, HKs were considered to be restricted to bacteria, but in the 1990s, HKs were identified in plants [2], fungi [3], archaea [4], cyanobacteria [5], and amoebae (Fig 1A) [6]. Soon after, evidence suggested that HKs regulate essential processes in pathogenic bacteria and fungi [7]. Although some HKs appear to be present in humans, typical bacterial or fungal HK-like sensor proteins have not been reported yet in mammals [8], promoting these proteins as ideal targets for future therapeutics [9].

It is now accepted that HKs are involved in cell signaling systems referred to as His-to-Asp phosphorelays and several canonical schemes depicting transduction pathways involving HKs in bacteria, amoebae, plants, and fungi have emerged (Fig 1B). To date, HKs act as primary sensors for various environmental stimuli, and, upon activation, initiate phosphate transfer events between various proteins, leading to an adaptive response. Although these mechanistic models are largely described in bacteria and plants, limited evidence is available for amoebae and fungi.

## Structure and Classification of Fungal Histidine Kinases

**Fig 2:**
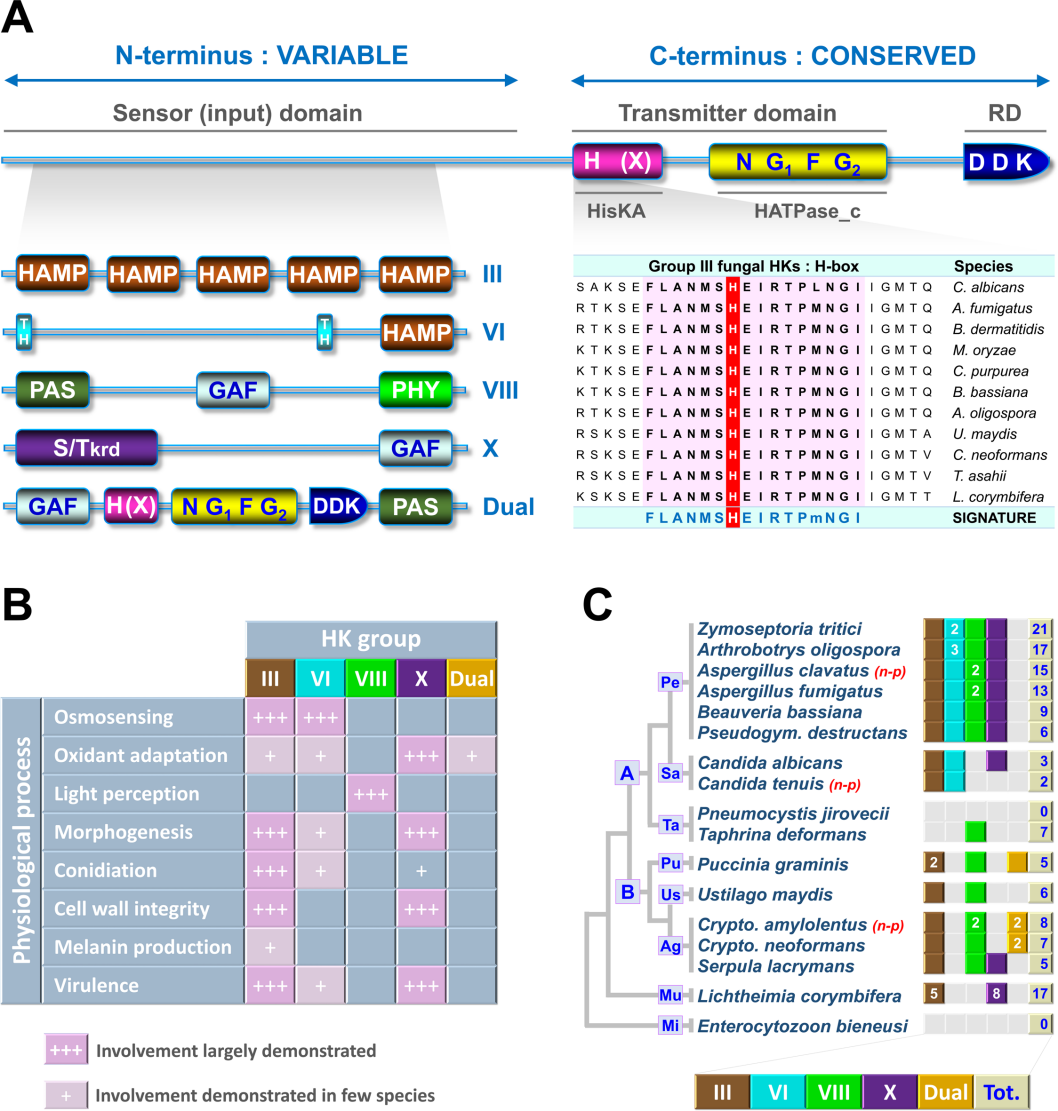
Structure, classification, function, and distribution of fungal HKs at a glance. (A) Basic structure of fungal HKs. They are composed of three main regions: a highly variable N-terminal sequence that determines which stimulus is perceived by the HK (“sensor” domain), a central transmitter domain consisting of both histidine kinase A (HisKA) and cognate histidine kinase-like ATPase catalytic subdomains (HATPase_c), and a C-terminal receiver domain showing a three amino-acids signature (DDK). Fungal HKs are currently categorized in 16 groups according to the sequence analysis of two regions: the H-box signature (alignment of group III HKs from major pathogenic fungi are provided in the right panel) containing the phosphorylatable histidine (red background) and the combination of domains found in the N-terminus. Domains that compose the N-terminal sensor region of major HK groups, whose functions have been at least partially characterized, are provided on the left panel. Abbreviations: HAMP, Histidine kinases-Adenylate cyclases-Methyl accepting proteins and Phosphatases; TH, Transmembrane Helix; PAS, Period circadian protein-Aryl hydrocarbon receptor nuclear translocator protein-Single-minded protein; GAF, cGMP-specific phosphodiesterases-Adenylyl cyclases-FhlA; PHY, Phytochrome; S/Tkrd, Serine/Threonine kinase related domain. (B) Some notable functions currently assigned to the prominent groups III, VI, VIII, X, and dual HKs (for more details see [11]). (C) Quantitative and qualitative distribution of HKs in fungal clades. The total number of HKs and the occurrence of major HK groups are provided for a panel of representative well-known pathogenic and non-pathogenic (n-p) fungi. A grey box signifies that the corresponding HK groups are not observed in the species. A colored box signifies that a unique member of the corresponding HK group is found in the species and the number of members is only indicated when many members are observed. Abbreviations: A, Ascomycota; B; Basidiomycota; Pe, Pezizomycotina; Sa, Saccharomycotina; Ta, Taphrinomycotina; Us, Ustilaginomycotina, Pu, Pucciniomycotina; Ag, Agaricomycotina; Mu, Mucoromycotina; Mi, Microsporidia.

The basic structure of fungal HKs is now well established. They are composed of three main regions (Fig 2A). The first region corresponds to a highly variable N-terminal sequence that determines which stimulus is perceived by the HK. This region is referred to as the “sensor domain.” The central region is the transmitter domain consisting of both histidine kinase A (HisKA) (dimerization/phosphoacceptor) (Fig 2A) and cognate histidine kinase-like ATPase catalytic (HATPase_c) (Fig 2A) subdomains. HisKA domains include an H-box, usually containing a phosphorylatable histidine (see Fig 2A), and an X-box. The HATPase_c subdomain displays four distinct boxes: N-, G1- (sometimes called D-box), F-, and G2-boxes. The third and final region, well conserved in fungal HKs, is the C-terminal receiver domain (RD) (Fig 2A) characterized by the presence of a three amino-acids signature (DDK) including a phosphorylatable aspartate residue (see Fig 2A) [10]. Another important aspect is that fungal HKs are generically defined as hybrid HKs since the transmitter domain is fused to the receiver domain (Fig 1B).

Sequence analysis of the transmitter domain and the sensor domain have aided in categorizing fungal HKs into 16 groups [11]. More precisely, the H-box signature (pink region in Fig 2A) containing the phosphorylatable histidine (red residue in Fig 2A) is often sufficient to classify an HK into its respective group [12]. However, since several fungal HK groups are divergent [12], it is essential to verify that the N-terminus of a newly identified HK contains most of the domains commonly found in HKs already assigned to the corresponding group. Thus, this means that each fungal HK group is defined by a relatively well-conserved H-box signature and a specific combination of domains within the N-terminus. For instance, the extensively studied group III fungal HKs are characterized by both a highly conserved H-box signature (alignment of group III HKs from prominent pathogenic species is provided in the Fig 2A) and tandem repeats of Histidine kinases-Adenylate cyclases-Methyl accepting proteins and Phosphatases (HAMP) domains within the N-terminus. The combination of domains that compose sensor region of functionally well-characterized HK groups (see following section) is provided in Fig 2A.

## Functions Currently Assigned to Fungal Histidine Kinases

HKs are key signaling proteins involved in the perception and the transduction of a wide range of environmental stimuli in prokaryotes, amoebae, and plants. HKs are also widespread in the fungal kingdom, but their precise roles in the regulation of physiological processes remain fragmentary. The function of several fungal HKs has been studied over the last 15 years using classical reverse genetic approaches, mainly by creating targeted mutants and comparing them with the wild type strain (for a review see [11]). This approach has given insight into the involvement of some fungal HK groups in the response and adaptation to environmental conditions. The most striking associations reported between fungal HKs and physiological processes are summarized below and in Fig 2B.

Some fungal HKs regulate osmotic adaptation by governing the high-osmolarity glycerol (HOG) mitogen-activated protein kinase (MAPK) pathway (Fig 1B). This includes members of HK groups VI and III (Fig 2A). Deletion of these groups of HKs may cause cell lethality not because of the absence of the osmosensing function, but because of hyperactivation of the HOG pathway causing overaccumulation of intracellular glycerol levels [13,14].

Some other fungal HKs display a key role in response to oxidative stress. This function is particularly attributed to the group X HKs (Fig 2A) but was also reported for a few HKs belonging to other groups. Interestingly, the group X HK members in pathogenic fungi that were initially found to be involved in chemical oxidant adaptation also protected the fungal cells against phagocytic cells of their host [15].

Fungal phytochromes are light-perceiving HK receptors, as is the case in bacteria and cyanobacteria but not in plants, where phytochromes curiously evolved for serine/threonine kinase activity [16]. These proteins commonly belong to group VIII (also called the Fph group) (Fig 2A) and aid red-light sensing in Ascomycota and Basidiomycota while other types of photoreceptors mediate shorter wavelength light sensing (for a review see [17]). Notably, it has been recently reported that a phytochrome HK in *Aspergillus nidulans*, FphA, modulates sexual differentiation through the HOG MAPK pathway by sensing red-light [18].

Adding to the aforementioned chemical and physical stressors/stimuli that can be perceived by HKs, it is important to remember that a number of HKs were also associated with essential fungal developmental programs. Specifically, some HK deletion strains, notably from group III or X HKs (and to a lesser extent group VI), displayed strong physiological perturbations, such as altered hyphal growth and reduced conidiation in pathogenic fungi and morphogenetic switch inability in dimorphic fungal pathogens. For instance, while a *Candida albicans* group X HK mutant strain is unable to switch from yeast to hyphae, *Blastomyces dermatitidis*, *Histoplasma capsulatum*, or *Penicillium marneffei* group III HK mutant strains are incapable of the hyphae to yeast transition. Furthermore, deletion of group III HK also diminishes filamentous growth and mating efficiency in *Cryptococcus neoformans*. It is important to point out that these morphogenetic perturbations seen in mutants are often associated with an alteration in cell wall composition or integrity resulting in a reduced virulence [19].

Finally, some other interesting roles were reported for a few series of fungal HKs, but they currently appear species-specific including melanin production, adaptation to hypoxia, regulation of secondary metabolism, and biofilm formation (for a review see [11]).

## Distribution of Histidine Kinases in the Fungal Kingdom

HKs are widespread in all fungal clades with the exception of Pneumocystidiomycetes (e.g., *Pneumocystis jirovecii*, the causal agent of pneumocystosis, Fig 2C) and microsporidia (e.g., *Enterocytozoon bieneusi*, a causal agent of microsporidiasis, Fig 2C). In light of new genomic data, the initial conviction that Ascomycota harbor the largest number of HKs was recently revised. Surprisingly, some previously unexplored early diverging fungal lineages, such as Mucoromycotina (e.g., the fungal pathogen *Lichtheimia corymbifera*, an agent of human mucormycosis, Fig 2C) and its closely clade Entomophtoromycotina (e.g., *Basidiobolus sp*. and *Conidiobolus sp*., agents of human entomophtoromycosis), includes species with about 15–20 predicted HKs [11]. Nevertheless, it is accepted that Ascomycete yeasts (e.g., *Candida albicans*, Fig 2C) contain fewer HKs than filamentous Ascomycete species (Fig 2C), in which the number of HKs appears particularly variable. Some filamentous Ascomycete families have species that display a large series of HKs, such as Dothideomycetes (e.g., the plant pathogen *Zymoseptoria tritici*, Fig 2C) or Orbiliomycetes (e.g., the nematode trapping fungus *Arthrobotrys oligospora*, Fig 2C). However, it is important to note that even within a family, the total number of HKs can drastically differ between species. For example, in Sordariomycetes, *Fusarium verticillioides* (a pathogen of maize producing deadly mycotoxins) encodes 16 HKs [12], whereas only 9 HK-encoding genes are present in the genome of *Beauveria bassiana* (a prominent insect pathogen, Fig 2C) [11]. Even more strikingly, in Leotiomycetes, *Botrytis cinerea* (the cause of the gray mold on many plants) encodes 20 HKs [12], whereas *Pseudogymnoascus destructans* (the causal agent of the white-nose syndrome in bats, Fig 2C) contains only 6 predicted HKs.

A recent compilation/classification based on genomic analysis of 50 species indicates that Basidiomycota species contains few (2 to 7) HK members and there is no marked trend between yeast and filamentous species from this clade [20]. For instance, the Agaricomycete yeast *C*. *neoformans* encodes 7 HKs, whereas 5 genes are predicted to encode HKs in *Serpula lacrymans*, a devastating wood degrading filamentous fungus from the same clade (Fig 2C).

Finally, major qualitative points concerning HK proteins found in Ascomycota, Basidiomycota, and other early diverging fungal lineages also require particular mention. Some HK groups are widely distributed among fungal clades (Fig 2C). This is the case of group III HKs, which are present in most of species that contain HKs with the exception of Taphrinomycotina (e.g., *Taphrina deformans*, the causal agent of peach leaf curl, Fig 2C), the Schizosaccharomycetaceae (e.g., the model fission yeast *Schizosaccharomyces pombe*), and the Saccharomycetaceae Whole Genome Duplication (WGD) clade (*e*.*g*. *Candida glabrata*). Interestingly, this observation has led some research teams to use group III HK-expressing strains of *Saccharomyces cerevisiae* as cell-based reporters to develop high throughput screening for new antifungals (see [9] for a review). On the other hand, some HK groups seem to have emerged or been maintained in a clade-specific manner. This has been hypothesized for the dual HK group that only occurs in Basidiomycota [21], or group VI osmosensors, which appear to be restricted to Ascomycota. Finally, it remains important to note that the broad series of HKs (up to 15) observed in some clades/species originates mainly from gene duplication events (likely leading to functional redundancy) and, to a lesser extent, from acquisition of new structures (HK groups). This is particularly obvious for the HKs found in Mucoromycotina (e.g., *L*. *corymbifera*) (Fig 2C) and filamentous Ascomycete plant pathogens [12].

In conclusion, it is striking to observe such a high degree of diversity among HKs in different fungi, both in the number of genes and their structure and functional variations, but, unfortunately, the underlying mechanisms that drive such diversity remain unclear. Closely related pathogenic and non-pathogenic fungal species do not appear to have significantly varying number of HKs (Fig 2C). However, it is highly likely that this gene family may have undergone rapid gene expansion under certain selection pressures allowing fungal species to adapt to their specific niches.

## Future Prospects in Fungal Histidine Kinase Research

Many functional and evolutionary aspects concerning HKs in the fungal kingdom are still largely uncharted. However, we can assume that the recent expansion of genomic resources along with improved genetic approaches for studying pathogenic fungi (see [22] for a review) will contribute to broadened knowledge about fungal HKs. It is likely that the availability of new genome sequences will aid in identifying unknown HK structures/groups, which will help complete the current classification of this protein family. In the same way, comparative genomics should also expedite comprehensive phylogenetic analyses, enabling us to decipher the evolutionary path that led to the appearance, transfer, duplication, and loss of HK genes in fungi. As recently observed for few species [23,24], the development of novel efficient gene disruption strategies now promotes systematic multiple gene deletions in molds that bear a large number of HKs. In the near future, these powerful genetic approaches will likely help in deciphering (1) the role of each HK in a species, (2) putative genetic interactions, and (3) the basis of their apparent functional redundancy. In addition, although basic HK-mediated signal transduction routes are well characterized, downstream interacting partners and the global architecture of the signaling pathway involving fungal HKs remain critical missing pieces in this field. For instance, fluorescence microscopy technics can provide insight into the subcellular localization of fungal HKs [25]. Notably, with the exception of group VI HKs harboring transmembrane regions, most HKs are not predicted to be localized on the cell surface. Therefore, it will be intriguing to study how an environmental signal is translated into intracellular input signal for intracellular HKs and even a possibility that HKs may collaborate with other sensor proteins. Additionally, it will be highly relevant to determine which of the known HK groups directly communicates with the only currently known downstream candidate, the phosphotransfer shuttle protein Ypd1 (see Fig 1B), and to identify unknown downstream interacting partners. It is puzzling how different signals coming from different HKs are converged into Ypd1 and a few (1 to 2) response regulators, implying that some adaptor or scaffold proteins, hitherto uncharacterized, may play such roles. Overall, we can assume that all the aforementioned strategies and outlooks will accelerate acquisition of new basic knowledge concerning fungal HK properties and general knowledge about signaling pathways involving these proteins. This is of primary importance for the future development of innovative HK-targeted antifungal strategies.
